# Relationship between Iron deposition and T lymphocytes in children with β-thalassemia with haematopoietic stem cell transplantation

**DOI:** 10.3389/fped.2022.939157

**Published:** 2022-10-17

**Authors:** Yuhang Zhou, Jianming Luo

**Affiliations:** Department of Paediatrics, First Affiliated Hospital of Guangxi Medical University, Nanning, China

**Keywords:** Iron deposition, T lymphocyte, β-thalassemia, haematopoietic stem cell transplantation, children

## Abstract

**Background:**

β-Thalassemia cellular immunity is associated with iron overload. However, the relationship between varying degrees of iron deposition and T cell immune recovery after allogeneic haematopoietic stem cell transplantation(allo-HSCT) in children remain unclear.

**Methods:**

A retrospective analysis was performed on 84 children with β-Thalassemia undergoing sibling allo-HSCT. According to the degrees of hepatic iron deposition, patients were divided into four classes. T lymphocyte counts were measured. Hepatic iron deposition was assessed by T2* MRI. Epstein–Barr virus and cytomegalovirus infection rates and graft-vs.-host disease incidence were recorded.

**Results:**

Immune recovery after allo-HSCT was compared between the two groups. Normal vs. mild group: CD4 cells were higher at 1, 3, and 6 months (*P* < 0.05), CD3 and CD8 cells were higher at 3 and 6 months, and 1 year in normal group (*P* < 0.05). Normal vs. moderate group: CD3 and CD4 cells were higher at 1, 3 and 6 months, and 1 year (*P* < 0.05), CD8 cells were higher at 1 and 3 months, and 1 year in normal group (*P* < 0.05). Normal vs. severe group: CD3, CD4 and CD8 cell at 1, 3 and 6 months, and 1 year in normal group (*P* < 0.05). Mild vs. moderate group: CD3, CD4 and CD8 cells were higher at 1 month in mild group (*P* < 0.05). Mild vs. severe group: CD4 cells were higher at 1, 3 and 6 month, and 1 year (*P* < 0.05), CD3 and CD8 cells were higher at 1 month in mild group (*P* < 0.05). Moderate vs. severe group: CD4 cells were higher at 3 months (*P* < 0.05), CD8 cells were higher at 6 months in moderate group (*P* < 0.05). The hepatic T2* values were positively correlated with CD3, CD4 and CD8 cells. The infection rates of Epstein-Barr virus and cytomegalovirus were significantly different among the groups (*P* < 0.05).

**Conclusion:**

Iron deposition affects immune recovery of T lymphocytes after allo-HSCT in children with β-thalassemia. The lower the levels of iron deposition, the greater the CD4 cell count.

## Introduction

β-Thalassemia (β-TM) is an autosomal recessive disorder characterized by β-reduced or absent globin chain synthesis. The imbalance in the number of alpha-globin and β-globin chains leads to early apoptosis of mature nucleated red cells, eventually causing chronic haemolytic anaemia (1). Due to repeated blood transfusions and increased intestinal iron absorption, the body lacks the route to excrete excessive iron, which eventually leads to iron deposition in multiple tissues, resulting in parenchymal organ damage (2). Allogeneic haematopoietic stem cell transplantation(allo-HSCT) can cure β-TM (3). However, HSCT is a challenging procedure with fatal complications, such as severe infection, graft-vs.-host disease (GVHD)and transplant rejection. These fatal complications are closely associated with immune system recovery after HSCT (4, 5). Therefore, immune system recovery after HSCT in β-TM patients needs to be better understood to improve overall survival and thalassemia-free survival.

In recent years, increasing attention has been given to damage to the immune system caused by iron overload after stem cell transplantation (6). Studies have found that iron overload can influence the number and function of T lymphocytes by increasing reactive oxygen species (7), thereby affecting cellular immunity in children with β- TM (8). Iron overload in β-TM results from ineffective erythropoiesis and repeated transfusions, ultimately leading to iron deposition in multiple organs, of which hepatic iron deposition is the most common. However, whether different levels of hepatic iron deposition affect the recovery of immune function is unclear. Therefore, in this study, we investigated the relationship between T lymphocyte reconstitution with different degrees of hepatic iron deposition after allo-HSCT.

## Materials and methods

### Study subjects

For this study, we retrospectively analysed the clinical data collected from patients who underwent sibling allo-HSCT at the Department of Paediatrics, the First Affiliated Hospital of Guangxi Medical University, between October 2017 and December 2020. The following inclusion criteria were employed: (1) The diagnosis was paediatric β-TM; (2) Donor-recipient pairs were human leukocyte antigen (HLA)-matched at the allele level (HLA-A, HLA-B, HLA-C, HLA-DRB1, HLA-DQB1, and HLA-DPB1); (3) Cardiac and hepatic MRI T2* values assessment was performed before allo-HSCT; (4) No iron chelation therapy within 1 year after transplantation. The following exclusion criteria were employed: (1) Children with graft failure; (2) Follow-up less than 1 year. The study subjects were divided into four groups according to hepatic MRI T2* values (9): no hepatic iron deposition group (normal), hepatic mild iron deposition group (mild), moderate hepatic iron deposition group (moderate), and severe hepatic iron deposition group (severe).

### Methods

#### Haematopoietic stem cell transplantation procedure

All patients underwent 9 days of myeloablative conditioning before transplantation. The conditioning regimen consisted of fludarabine for 3 days (50 mg/m^2^/day; days −9 to −7), busulfan for 4 days (4 mg/kg/day; days −9 to −6), cyclophosphamide for 4 days (50 mg/kg/day; days −5 to −2), and antithymocyte globulin for 4 days (2.5 mg/kg/day; days −5 to −2). All children were treated with hydroxyurea (20–50 mg/kg/day) orally for 3–6 months before transplantation. All children were treated with cyclosporine and methylprednisolone to prevent GVHD.

#### Definitions

Engraftment was defined as the first of 3 consecutive days when the absolute neutrophil count was ≥0.5 × 10^9^/L and the absolute platelet count was ≥20 × 10^9^/L without granulocyte colony stimulating factor and transfusion. Diagnostic criteria for GVHD were based on the National Institutes of Health consensus classification (10).

#### Iron deposition detection

The pre-transplant magnetic resonance imaging (MRI) of all patients was performed using a 3.0 T scanner (Verio, Siemens, Germany), and data were analysed using CMR Tools software (England). According to the hepatic and cardiac T2* values, iron deposition was divided into four classes (9). Cardiac: normal (T2* > 20 ms), mild (14 ms ≤ T2* < 20 ms), moderate (10 ms ≤ T2* < 14 ms), and severe (T2* < 10 ms). Hepatic: normal (T2* ≥ 6.3 ms), mild (2.6 ms ≤ T2* < 6.3 ms), moderate (1.4 ms ≤ T2* < 2.6 ms), and severe (T2* < 1.4 ms).

#### Lymphocyte subsets detection

Lymphocyte subsets were determined preoperatively and at 1, 3, 6, and 12 months postoperatively. Data were acquired on a BD FACSCantoTM II flow cytometer and analysed using BD FACSC Canto Software. Peripheral blood was collected in tubes containing EDTA. The BD Multitest ™ IMK kit was used. Cells were stained with mouse anti-human monoclonal antibody (CD45/CD4/CD8/CD3) recognizing CD3, CD4, CD8 and red blood cells lysed with erythrocyte lysing solution.

#### Routine laboratory testing

Routine laboratory testing included liver function, cardiac enzymes, electrocardiogram (ECG), and abdominal hepatic and cardiac ultrasound before HSCT. Epstein-Barr virus(EBV) and cytomegalovirus (CMV)infections were confirmed by peripheral blood PCR in the laboratory after HSCT.

### Statistical analysis

Date were performed using SPSS26.0 software. Continuous data are shown as the median (four-digit interval) [P50(P25, P75)]. Two-group comparisons were performed using the Mann–Whitney *U* test. Multiple groups were compared using the Kruskal–Wallis H test. Bonferroni test was used to compare differences for variables among groups. Paired samples were analyzed by Wilcoxon signed-rank test. Proportion data are presented as numbers (percentages). The Pearson Chi-Square, Corrected Chi-Square or Fisher's Exact test was used to perform multiple groups comparisons. Correlations between variables were analysed by Spearman. Two-sided *P* values <0.05 were considered significant for all analyses.

## Results

### Clinical characteristics of patients

Over the period of observation, a total of 95 children with β-TM who underwent sibling allo-HSCT were enrolled. Two children were excluded because of graft failures, and five was excluded because of death within 1 year after transplantation, and four was excluded because of loss to follow-up. Finally, 84 children were included in this study. The study subjects were divided into four groups: normal (*n* = 11), mild (*n* = 17), moderate (*n* = 17), and severe (*n* = 39). There were no significant differences in sex, age, weight, graft source, stem cell (CD34+) number and total number of nucleated cells among all groups (*P > *0.05). The clinical characteristics of the children are shown in Table 1.

**Table 1:** Group characteristics (*n* = 84).

Characteristic	Normal	Mild	Moderate	Severe	*P*
Subjects, *n*	11	17	17	39	—
Sex, male, *n* (%)	4 (36.4)	8 (47.1)	9 (52.9)	26 (66.7)	0.056
Age [year, P50 (P25, P75)]	8.23 (6.08, 9.41)	5.67 (4.34, 7.88)	5.25 (4.46, 8.25)	7.67 (5.08, 10.08)	0.280
Weight[kg, P50 (P25, P75)]	22.00 (18.00, 27.10)	18.30 (15.55, 23.75)	18.00 (15.00, 23.25)	21.00 (16.30, 26.00)	0.462
Graft source					
BM + PB, *n* (%)	5 (45.5)	11 (64.7)	10 (58.8)	21 (53.8)	0.766
BM + CB, *n* (%)	6 (54.5)	6 (35.3)	7 (41.2)	18 (46.2)	
Stem cell (CD34 +)/kg [ × 10^6^, P50 (P25, P75)]	6.33 (4.92, 9.96)	3.08 (1.31, 5.60)	6.19 (1.90, 8.40)	5.67 (3.10, 7.66)	0.062
Total number of nucleated cells/kg [ × 10^8^, P50 (P25, P75)]	12.65 (1.86, 22.82)	2.92 (1.15, 9.18)	4.04 (2.21, 15.72)	3.49 (1.72, 18.11)	0.091

BM, bone marrow. PM, peripheral blood. CB, cord blood.

### Comparison of T-lymphocyte subsets

Normal vs. mild group: The normal group had higher numbers of CD3 cells at 3 months, 6 months and 1 year after HSCT (*P* = 0.029, *P* = 0.000 and *P* = 0.004, respectively), and no significant differences were noted at pretransplantation or 1 month after HSCT (*P* = 0.589 and *P* = 0.290, respectively). The normal group had higher numbers of CD4 cells at 1, 3, and 6 months after HSCT (*P *= 0.045, *P *= 0.020 and *P *= 0.001, respectively), and no differences were noted at pretransplantation and 1 year after HSCT (*P* = 0.495 and *P* = 0.180, respectively). The normal group had higher numbers of CD8 cells at 3 and 6 months, and 1 year after HSCT (*P *= 0.043, *P *= 0.000 and *P *= 0.015, respectively), and no significant differences were observed at pretransplantation, 1 month after HSCT (*P* = 0.869 and *P* = 0.359, respectively).

Normal vs. moderate group: The normal group had higher numbers of CD3 cells at 1, 3 and 6 months, and 1 year after HSCT (*P *= 0.018, *P *= 0.003, *P *= 0.012 and *P *= 0.004, respectively), and no significant differences were noted pretransplantion (*P *= 0.869). The normal group had higher numbers of CD4 cells at 1, 3 and 6 months, and 1 year after HSCT (*P *= 0.003, *P *= 0.007, *P *= 0.000 and *P *= 0.036, respectively), and no differences were noted pretransplantion (*P* = 0.466). The normal group had higher numbers of CD8 cells at 1 and 3 months, and 1 year after HSCT (*P *= 0.036, *P *= 0.010 and *P *= 0.046, respectively), and no significant differences were observed at pretransplantion, 6 month after HSCT (*P* = 0.906 and *P* = 0.051, respectively).

Normal vs. severe group: The normal group had higher numbers of CD3 cells at pretransplantion, 1, 3 and 6 months, and 1 year after HSCT (*P *= 0.005, *P *= 0.000, *P *= 0.006, *P *= 0.000 and *P *= 0.001, respectively). The normal group had higher numbers of CD4 cells at pretransplantion, 1, 3 and 6 months, and 1 year after HSCT (*P *= 0.010, *P *= 0.000, *P *= 0.000, *P *= 0.000 and *P *= 0.002, respectively). The normal group had higher numbers of CD8 cells at pretransplantion, 1, 3 and 6 months, and 1 year after HSCT (*P *= 0.038, *P *= 0.002, *P *= 0.045, *P *= 0.000 and *P *= 0.010, respectively).

Mild vs. moderate group: The mild group had higher numbers of CD3 cells at 1 month after HSCT (*P *= 0.018), and no significant differences were noted at pretransplantion, 3 and 6 months, and 1 year after HSCT (*P *= 0.438, *P *= 0.326, *P *= 0.191 and *P *= 0.535, respectively). The mild group had higher numbers of CD4 cells at 1 month after HSCT (*P *= 0.033), and no differences were noted at pretransplantion, 3 and 6 months, and 1 year after HSCT (*P *= 0.986, *P *= 0.513, *P *= 0.352 and *P *= 0.168, respectively). The mild group had higher numbers of CD8 cells at 1 and 6 months after HSCT (*P *= 0.022 and *P *= 0.044, respectively), and no significant differences were observed at pretransplantion, 3 month and 1 year after HSCT (*P* = 0.438, *P* = 0.361 and *P* = 0.692, respectively).

Mild vs. severe group: The mild group had higher numbers of CD3 cells at pretransplantion and 1 month after HSCT (*P *= 0.010 and *P *= 0.000, respectively), and no significant differences were noted at 3 and 6 months, and 1 year after HSCT (*P *= 0.368, *P *= 0.880 and *P *= 0.073, respectively). The mild group had higher numbers of CD4 cells at pretransplantion, 1, 3 and 6 month, and 1 year after HSCT (*P *= 0.018, *P *= 0.000, *P *= 0.001, *P *= 0.019 and *P *= 0.016, respectively). The mild group had higher numbers of CD8 cells at pretransplantion, and 1 month after HSCT (*P *= 0.023 and *P *= 0.000, respectively), and no significant differences were observed at 3, 6 months and 1 year after HSCT (*P* = 0.656, *P* = 0.936 and *P* = 0.562, respectively).

Moderate vs. severe group: The moderate group had higher numbers of CD3 cells pretransplantion (*P *= 0.002), and no significant differences were noted at 1,3 and 6 months, and 1 year after HSCT (*P *= 0.762, *P *= 0.095, *P *= 0.055 and *P *= 0.273, respectively). The moderate group had higher numbers of CD4 cells at pretransplantion, and 3 month after HSCT (*P *= 0.018 and *P *= 0.019, respectively), and no differences were noted at 1 and 6 months, and 1 year after HSCT (*P *= 0.175, *P *= 0.066 and *P *= 0.092, respectively). The moderate group had higher numbers of CD8 cells at pretransplantion and 6 months after HSCT (*P* = 0.005 and *P *= 0.044, respectively), and no significant differences were observed at 1, 3 months and 1 year after HSCT (*P* = 0.895, *P* = 0.762 and *P* = 0.387, respectively). Comparison of T-lymphocyte subsets are summarized in Table 2.

**Table 2:** Comparison of T-lymphocyte subsets between groups before and after HSCT.

	Normal	Mild	Moderate	Severe
Before HSCT CD3 (cells/µl)	2018 (1920, 2685)	2084 (1718, 2345)	2183 (1824, 2723)	1676 (1248, 1957)^a^^b^^c^
Before HSCT CD4 (cells/µl)	1023 (930, 1665)	1064 (861, 1225)	1088 (862, 1308)	830 (557, 1174)^a^^b^^c^
Before HSCT CD8 (cells/µl)	877 (773, 1094)	851 (699, 1045)	891 (750, 1079)	603 (493, 945)^a^^b^^c^
1 month CD3 (cells/µl)	270 (147, 584)	232 (166, 289)	110 (26, 214)^a^^b^	107 (35, 166)^a^^b^
1 month CD4 (cells/µl)	69 (33, 102)	42 (20, 64)^a^	16 (9, 41)^a^^b^	14 (4, 18)^a^^b^
1 month CD8 (cells/µl)	219 (109, 392)	162 (130, 214)	77 (17, 173)^a^^b^	80 (22, 141)^a^^b^
3 month CD3 (cells/µl)	919 (795, 1221)	704 (492, 821)^a^	602 (469, 725)^a^	537 (396, 821)^a^
3 month CD4 (cells/µl)	186 (123, 289)	132 (110, 155)^a^	117 (90, 179)^a^	98 (47, 108)^a^^b^^c^
3 month CD8 (cells/µl)	723 (557, 912)	493 (359, 623)^a^	440 (320, 527)^a^	402 (328, 641)^a^
6 month CD3 (cells/µl)	1459 (1302, 2695)	1048 (690, 1262)^a^	1200 (922, 1706)^a^	897 (721, 1320)^a^
6 month CD4 (cells/µl)	432 (389, 488)	316 (227, 403)^a^	298 (219, 373)^a^	259 (140, 306)^a^^b^
6 month CD8 (cells/µl)	1030 (895, 1912)	739 (311, 814)^a^	852 (615, 1383)^b^	644 (417, 745)^a^^c^
1 year CD3 (cells/µl)	3545 (2811, 4477)	2131 (1758, 2696)^a^	1934 (1394, 2384)^a^	1697 (1213, 2109)^a^
1 year CD4 (cells/µl)	893 (728, 1618)	853 (588, 1261)	764 (588, 946)^a^	693 (455, 813)^a^^b^
1 year CD8 (cells/µl)	1702 (1201, 3706)	1099 (735, 1215)^a^	1108 (697, 1402)^a^	873 (585, 1168)^a^

HSCT, haematopoietic stem cell transplantation.

^a^ Means when compared with the normal group, *P* < 0.05.

^b^ Means when compared with the mild group, *P* < 0.05.

^c^ Means when compared with the moderate group, *P* < 0.05.

### Correlation of hepatic T2* values with T lymphocytes

**Figure 1:**
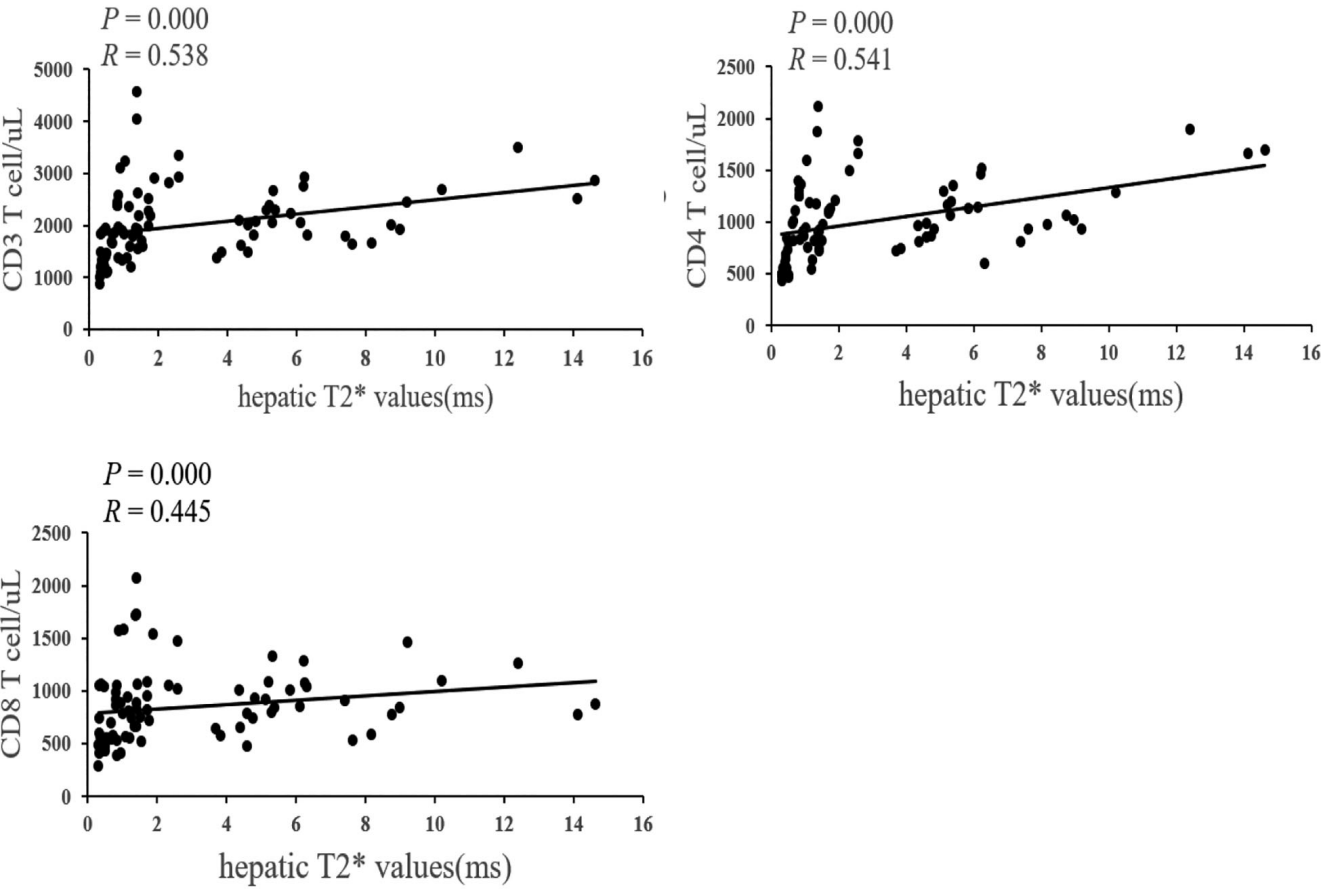
Correlation between hepatic T2* values and CD3, CD4, CD8 cells..

The hepatic T2* values were positively correlated with CD3, CD4 and CD8 cells (*P* = 0.000 and R = 0.538, *P* = 0.000 and R= 0.541, *P* = 0.000 and R = 0.445) (Figure 1).

### Comparison of EBV and CMV infection rates among groups after HSCT

EBV infection occurred in 1 patient (5.9%) in the mild group, 2 patients (11.8%) in the moderate group, and 13 patients (33.3%) in the severe group within 1 year of HSCT. The normal group did not show EBV infection. The incidence of EBV was significantly different among the groups, as shown in Table 3.

**Table 3:** Incidence of infection and GVHD in each group after HSCT.

	Normal	Mmild	Moderate	Severe	*P*
EBV *n*, (%)	0	1 (5.9)	2 (11.8)	13 (33.3)	0.020
CMV *n*, (%)	4 (26.7)	4 (23.5)	9 (52.9)	25 (64.1)	0.002
aGVHD *n*, (%)	3 (27.3)	2 (11.8)	1 (5.9)	6 (15.4)	0.570
Grade I *n*, (%)	2 (18.2)	2 (11.8)	0	4 (10.3)	
Grade II *n*, (%)	1 (9.1)	0	1 (5.9)	2 (5.1)	
cGVHD *n*, (%)					
Moderate	0	0	0	1 (2.6)	

EBV, epstein–barr virus, CMV, cytomegalovirus, GVHD, graft-versus-host disease. HSCT, haematopoietic stem cell transplantation.

CMV infection occurred in 4 cases (26.7%) in the normal group, 4 cases (23.5%) in the mild group, 9 cases (52.9%) in the moderate group, and 25 cases (64.1%) in the severe group within 1 year after HSCT. The incidence of CMV was significantly different among the groups (*P = *0.002), as shown in Table 3.

EBV and CMV infections were treated with ganciclovir or valganciclovir, and most patients had a favourable prognosis. One EBV patient developed lymphoproliferative disease. By reducing the immunosuppressant dose, rituximab and ganciclovir therapy, the patient was cured.

### Comparison of GVHD incidence among groups after HSCT

In the normal group, 3 patients (27.3%) had acute GVHD (aGVHD) after HSCT, including 2 with skin aGVHD (grade I), and 1 with skin aGVHD (grade I) combined with gastrointestinal aGVHD (grade II). In the mild group, 2 patients (11.8%) had skin aGVHD (grade I) after HSCT. In the moderate group, 1 patients (5.9%) had skin aGVHD (grade II) after HSCT. In the severe group, 7 patients (17.9%) had GVHD after HSCT, including 4 with skin aGVHD (grade I), 1 with skin aGVHD (grade I) combined with liver aGVHD (grade II), 1 with liver aGVHD (grade II), and 1 with moderate liver cGVHD. No significant difference in the incidence of aGVHD was noted among the groups (*P* = 0.570), as shown in Table 3.

### Liver function and structure

Liver function was compared among multiple groups, and differences in the levels of total bile acids (TBA) and alanine aminotransferase (ALT) were statistically significant (*P* = 0.034 and *P* = 0.005, respectively). There were significant differences in TBA between normal and mild groups(*P* = 0.031). The ALT level in the severe group was significantly different from those in the normal and mild groups (*P* = 0.033 and *P* = 0.023, respectively). According to hepatic ultrasound results, 2 patients (18.2%) had hepatomegaly in the normal group, 6 patients (35.3%) in the mild group, 9 patients (52.9%) in the moderate group, 23 patients (59.0%) in the severe group. Hepatomegaly was no significant difference among the groups (*P* = 0.070). The data are summarized in Table 4.

**Table 4:** Comparison of liver structure and function.

Characteristic	Normal	Mild	Moderate	Severe	*P*
TBIL[µmol/L, P50 (P25, P75)]	16.70 (9.30, 28.30)	15.20 (10.90, 28.30)	14.10 (9.10, 17.70)	15.60 (10.20, 23.50)	0.721
DBIL[µmol/L, P50 (P25, P75)]	5.20 (3.20, 6.60)	2.70 (2.70, 5.80)	3.30 (2.30, 5.10)	3.30 (2.50, 5.60)	0.686
IBIL[µmol/L, P50 (P25, P75)]	11.30 (6.10, 16.40)	11.50 (8.10, 20.90)	10.60 (6.90, 14.30)	11.20 (6.90, 16.40)	0.720
TBA[µmol/L, P50 (P25, P75)]	5.50 (3.60, 6.60)	13.00 (8.22, 16.00)^a^	19.50 (5.80, 16.00)	8.70 (5.72, 14.10)	0.034
AST [U/L, P50 (P25, P75)]	24.00 (19.00, 30.00)	27.00 (20.05, 33.50)	30.00 (25.50, 38.00)	32.00 (22.00, 51.00)	0.640
ALT[U/L, P50 (P25, P75)]	14.00 (10.00, 26.00)	16.00 (12.00, 22.50)	20.00 (16.50, 47.00)	35.00 (16.00, 59.00)^a^^b^	0.005
Hepatomegaly *n*, (%)	2 (18.2)	6 (35.3)	9 (52.9)	23 (59.0)	0.070

TBIL, total bilirubin. DBIL, direct bilirubin. IBIL, indirect bilirubin. TBA, total bile acid. AST, Aspartate aminotransferase. ALT, Alanine aminotransferase.

^a^ Means when compared with the normal group, *P* < 0.05.

^b^ Means when compared with the mild group, *P* < 0.05.

### Cardiac function and structure

Thirteen patients (15.6%) exhibited cardiac iron deposition; 8 patients had mild iron deposition, 4 patients had moderate iron deposition, and 1 patient had severe iron deposition. These cases were complicated by different degrees of hepatic iron deposition (mild *n* = 2, moderate *n* = 4, severe *n* = 7). Compared with the normal group, creatine kinase(CK), creatine kinase isoenzyme(CK-MB) and lactate dehydrogenase(LDH) were no significantly different(*P* = 0.772, *P* = 0.581 and *P* = 0.706, respectively). In the cardiac iron deposition group, 8 (61.5%) patients exhibited an abnormal cardiac structure: 5 patients had left ventricle enlargement and 3 patients had left ventricle and atrium enlargement. Six (46.2%) patients had ECG abnormalities, 4 patients had QT interval prolongation, 1 patient exhibited a high voltage of the left ventricle, and 1 patient had ST-segment elevation. In the normal group, 3 (27.3%) patients showed an abnormal cardiac structure: 1 patient had left ventricle enlargement and 2 patients had left ventricle and atrium enlargement. 5(45.5%) patients had ECG abnormalities, 3 patients had QT interval prolongation, 2 patitents had ST-segment elevation. Cardiac structural and ECG abnormalities did not differ significantly between the cardiac iron deposition and normal groups(*P* = 0.123 and *P* = 1.000, respectively). The data are summarized in Table 5.

**Table 5:** Comparison of cardiac structure and function.

Characteristic	Normal	Cardiac iron deposition	*P*
CK[U/L, P50 (P25, P75)]	59.00 (39.00, 94.00)	48.00 (38.00, 80.50)	0.772
CK-MB[U/L, P50 (P25, P75)]	13.00 (10.00, 19.00)	14.00 (11.00, 19.50)	0.581
LDH[U/L, P50 (P25, P75)]	233.00 (185.00, 256.00)	223.00 (200.50, 276.50)	0.706
Abnormal cardiac structure *n*, (%)	3 (27.3)	8 (61.5)	0.123
Abnormal electrocardiogram *n*, (%)	5 (45.5)	6 (46.2)	1.000

CK, creatine kinase; CK-MB, creatine kinase MB isoenzyme; LDH, lactate dehydrogenase.

### Cardiac and hepatic T2* values

Only 21 patients completed MRI examination 1 year after HSCT without undergoing iron chelation therapy before examination. The median cardiac T2* values of these 21 patients were 18.28 ms before HSCT and 21.11 ms 1 year after HSCT. There was no significant difference in cardiac T2* values before and after HSCT (*P* = 0.487). The median hepatic T2* values of these 21 patients were 1.86 ms before HSCT and 1.41 ms 1 year after HSCT. Hepatic T2* values before HSCT were higher than those at 1 year after HSCT (*P* = 0.002). The data are summarized in Table 6.

**Table 6:** T2* MRI values before and after HSCT.

	Before allo-HSCT	1 year	*P*
hepatic T2* values (ms)	1.86 (1.17, 5.03)	1.41 (1.02, 3.39)	0.002
Cardiac T2* values (ms)	18.28 (12.50, 22.78)	21.11 (13.40, 26.01)	0.487

HSCT, haematopoietic stem cell transplantation.

## Discussion

Allo-HSCT refers to receiving multipotent haematopoietic stem cells to achieve haematopoietic and immune reconstruction. This medical procedure has been widely used to treat gene defect diseases, solid tumours, malignant haematopoietic diseases, and autoimmune and congenital diseases. Haematopoietic reconstruction after transplantation is often regarded as a marker of successful engraftment. However, immune function reconstitution requires more time, and considerable interindividual variation has been observed in this reconstitution. The recovery of immune function closely relates to the prognosis of HSCT. Delayed immune reconstitution may increase the risk of disease recurrence, viral infection and secondary malignancy (5). Therefore, the regular monitoring of immune function reconstitution after HSCT with corresponding interventional therapy is important to reduce post-transplant infection and tumour recurrence. Immune recovery after HSCT is influenced by multiple factors, including the degree of human leukocyte antigen match between the donor and recipient, the conditioning regimen before transplantation, the use of immunosuppressive agents after transplantation, and the presence of infections in the donor and recipient before transplantation. In addition to the abovementioned factors, the current work focuses on the effect of iron on immune recovery after transplantation. Iron overload can disturb the immune balance, supporting the growth of infectious microorganisms (11). It also produces oxygen free radicals, leading to peroxidative damage to tissues and accelerated ageing of the immune system, followed by a progressive decline in reactivity to antigens and abnormal T cell function (12, 13). Iron overload also affects the balance between helper T cells and cytotoxic T cells and affects their proliferation (14). Several studies have shown that iron overload is associated with impaired immune responses in thalassemia patients (15, 16). Kadam et al (17). found that iron overload in children with β-TM led to decreased CD4 and CD8 T cell levels. Excess iron is deposited in various organs and tissues, such as the liver, heart, and pancreas. Hepatic iron deposition is most common in β-TM. MRI-T2* has been considered the gold-standard method for the detection iron deposition in different organs such as the hepatic and cardiac cardiac (18, 19). However, it is unclear whether different levels of hepatic iron deposition affect the recovery of immune function. The 84 patients were divided into four groups based on the extent of hepatic iron deposition on MRI T2* values, investigated the relationship between T lymphocyte reconstitution with different degrees of hepatic iron deposition after allo-HSCT. In this sdudy, at 1, 3, and 6 months and 1 year after HSCT, CD4 T cell counts were higher in the normal group than in the mild, moderate and severe iron deposition groups. At 1, 3, and 6 months and 1 year after HSCT, the number of CD4 T cells in the mild group were higher than in the moderate iron deposition group. At 3 months after transplantation, the number of CD4 T cells in the moderate group were higher than in the severe iron deposition group. Therefore, we speculated that iron deposition may be closely related to the CD4 T cell counts. Accordingly, a Spearman correlation analysis revealed that hepatic T2* values were positively correlated with CD4 T cell counts (*P* = 0.000, R= 0.541). For CD8 T cell counts, at 1 month after HSCT, it was higher in the normal and mild group than in the severe iron deposition group. At 3 months and 1 year after HSCT, it was higher in the normal group than in the mild, moderate and severe iron deposition group. At 6 months after HSCT, it was higher in the moderate group than in the severe group. we similarly performed Spearman correlation analysis and found that hepatic T2* values were positively correlated with CD8 T cell counts (*P* = 0.000 and R = 0.445). For CD3 T cell counts, at 1, 3, and 6 months and 1 year after HSCT, it was higher in the normal group than in the mild, moderate and severe iron deposition group. In the 1 month after HSCT, it was higher in the mild than in the moderate and severe group. We also found a positive correlation between hepatic T2* values and CD3 T cell counts (*P* = 0.000 and *R* = 0.538). In summary, we inferred that the lower the levels of iron deposition, the greater the CD4 cell counts.

T lymphocytes play an important role in the immune system. T cells are both important players in cellular immunity and regulators of humoral immunity. T lymphocytes are primarily responsible for cellular immunity to combat tumours and microorganisms as well as transplant rejection. T lymphocytes can be mainly subdivided into three subsets based on their specific immune effects: helper T cells, cytotoxic T lymphocytes and regulatory T lymphocytes. In addition, according to the presence of different surface proteins, T lymphocytes may also be divided into CD4 and CD8 T cells. Helper T cells are known as CD4 T lymphocytes. CD4 T lymphocytes can recruit neutrophils to the site of infection by secreting cytokines to help fight invading pathogens. CD4 T lymphocytes can also licence dendritic cells to induce the activation of CD8 T cells, perhaps with a direct cytotoxic function. Furthermore, these cells are also critical for antibody class switching. Based on these biological features, CD4 T cells can provide strength to fight against acute and chronic viral infections. The CD8 molecule, a leukocyte differentiation antigen, is a glycoprotein on the surface of a subset of T cells that assists T-cell receptors in recognizing antigens and participating in the transduction of T-cell activation signals, also known as coreceptors for T-cell receptors. CD8 T cells are known as cytotoxic T lymphocytes because they usually differentiate into cytotoxic T lymphocytes after activation and are able to specifically kill target cells. Therefore, high levels of CD4 and CD8 are associated with a reduced risk of developing infection.Our experimental data found that lower the levels of iron deposition, the greater the CD4 cell count. EBV and CMV are common viral infections after transplantation. After EBV infects the human body, cellular immunity plays a critical role in restricting and killing the virus, particularly cytotoxic T lymphocytes. After HSCT, immune function is significantly weakened, latent EBV is activated, and B lymphocytes transform to result in malignant proliferation that causes posttransplant lymphoproliferative disease (20). Post-transplant lymphoproliferative disease usually progresses rapidly and has a very high mortality rate. CMV infection is one of the most common complications after allo-HSCT, occurring in 30%–70% of patients (21). The clinical manifestations of CMV infection after allo-HSCT include pulmonary infection, gastric and intestinal inflammation, encephalitis, hepatitis, retinitis, and asymptomatic infection, among which CMV pulmonary infection is the most severe, with case fatality rates exceeding 50% (22). CMV infection and reactivation after transplantation not only increase the risk of infection with other bacteria and viruses but can also cause poor graft function or graft failure in recipients. Therefore, we compared the infection rates of EBV and CMV in different iron deposition groups. We found that the incidence of EBV(*P* = 0.020) and CMV (*P* = 0.002)was significantly different among the groups. EBV infection occurred in 0 case in the normal group, 1 case (5.9%) in the mild group, 2 cases (11.8%) in the moderate group, and 13 cases (33.3%) in the severe group within 1 year after HSCT. The normal group did not show EBV infection. CMV infection occurred in 4 cases (26.7%) in the normal group, 4 cases (23.5%) in the mild group, 9 cases (52.9%) in the moderate group, and 25 cases (64.1%) in the severe group within 1 year after HSCT. EBV and CMV infections were treated with ganciclovir or valganciclovir, and most patients had a favourable prognosis. One EBV patient developed lymphoproliferative disease. By reducing the immunosuppressant dose, rituximab and ganciclovir therapy, the patient was cured.

Allo-HSCT is one of the main therapeutic strategies for β-TM. However, GVHD is one of the major causes of transplant-related death. The mechanism underlying this transplant-related death is that antigen-presenting cells activate donor-derived allogenic reactive T cells, resulting in the massive expansion of the latter. These cells then attack the recipient's own tissues, resulting in tissue cell injury and organ dysfunction (23). Therefore, the occurrence of GVHD is related to immune function after allo-HSCT. Some studies have shown that iron overload can reduce the incidence of GVHD through its immunosuppressive effect (24–26). Studies on possible differences in the incidence of GVHD in patients with different degrees of hepatic iron deposition after HSCT are scarce. In this study, we found that there was 3 patients (27.3%) had aGVHD in the normal group, 2 patients (11.8%) had aGVHD in the mild group, 1 patients (5.9%) had aGVHD in the moderate group, 7 patients (17.9%) in the severe group, including 6 with aGVHD, 1 with liver cGVHD. There are no significant difference in the incidence of aGVHD was noted among the groups (*P* = 0.570), so it is necessary to expand the sample size for confirmation in the future. A total of 13 patients developed GVHD, 12 with acute GVHD and 1 with moderate liver cGVHD. This cGVHD patient developed hepatic rejection 11 months after HSCT. Clinical manifestation yellowish sclerae and hepatomegaly. liver function tests showed markedly elevated total bilirubin, TBA, transaminases and glutamyl transpeptidase(elevated total bilirubin but ≤3 mg/dl and alanine aminotransferase >5 ULN). Liver biopsy suggested chronic hepatitis and fibrosis. Oral cyclosporine were continued and adding methylprednisolone, the child improved and is still in follow-up. Acute GVHD patients were free. Only the moderate liver cGVHD patients still on immunosuppression at 1 year after HSCT.

β-TM is a genetic disorder characterized by defective synthesis of the β-globulin chain, which is mainly characterized by ineffective erythropoiesis and chronic haemolytic anaemia. Repeated blood transfusions and increased iron uptake from the gastrointestinal tract lead to iron deposition in the body. Without the timely administration of effective iron chelation therapy, this iron is widely deposited in the parenchymal cells of organs, such as the liver and heart, leading to the structural damage and dysfunction of these important organs. Iron deposition in the liver can lead to fibrosis or cirrhosis and may even lead to liver cancer (27). Iron deposition in the heart can cause cardiac dysfunction, arrhythmia and heart failure and is an important cause of death in thalassemia patients (28, 29). In this study, a total of 40 patients had hepatomegaly, hepatomegaly was no significant difference among the groups. For liver function, TBA and ALT were statistically significant among multiple groups. The Bonferroni test only found significant differences in the TBA between the normal and mild groups, and the ALT level in the severe group was significantly different from those in the normal and mild groups. Therefore, we inferred that hepatic iron deposition may affect liver function. There were 13 cases (15.6%) of cardiac iron deposition, all of which were complicated with different degrees of hepatic iron deposition. But there were no significant differences in cardiac function and stucture compared with the normal group. Perhaps because cardiac structure and function are affected not only by iron deposition but also by whether blood is transfused regularly.

Although HSCT successfully solved bone marrow defects in children with thalassemia, iron deposition exists for a long time after HSCT and could affect long-term survival rate (30). Therefore, adequate diagnosis and management of iron depositin after HSCT in various visceral organs is necessary (31). MRI-T2* has been considered the gold-standard method for the detection and quantification of iron deposition in different organs such as the hepatic and cardiac (18, 19). To better evaluate iron overload in patients, all patients in this study underwent complete cardiac and hepatic T2* testing by MRI before HSCT, and none were treated with chelation within 1 year after HSCT. One year after HSCT, only 21 patients underwent repeat MRI -T2* detection in cardiac and hepatic examinations on time. In these 21 patients, the cardiac T2* value was not significantly different before and after HSCT. However, the hepatic T2* value was decreased, and hepatic iron deposition was aggravated. This finding is consistent with that reported by Amir Ali Hamideh et al., who showed cardiac and hepatic iron deposition changes after HSCT in thalassemia patients (32). After HSCT, thalassemia patients obtain effective erythropoiesis. Phlebotomy is the preferred mechanism for removing excess iron after HSCT. Phlebotomy is effective, safe and inexpensive in both children and adults after HSCT (33, 34). However, due to the influence of children's compliance and venous access, performing a venotomy is difficult in patients under 11 years of age (35). The efficacy and safety of iron chelation in patients with thalassemia have been fully confirmed by using deferoxamine, deferiprone and deferasirox (36, 37). Due to the long subcutaneous injection time of deferoxamine, deferiprone has the risk of reducing neutrophils. For iron chelation therapy after transplantation, most studies have focused on the efficacy of deferasirox. A prospective randomized trial found that deferasirox was effective and safe in the treatment of iron burden in children with β-thalassaemia after HSCT (38). Therefore, deferasirox is recommended for paediatric patients who are not candidates for phlebotomy.

## Conclusion

Iron deposition can affect the immune recovery of T lymphocytes and increase rates of EBV and CMV infection after all-HSCT in children with thalassemia. The lower the levels of iron deposition, the greater the CD4 cell count. Therefore, MRI-T2* should be detected before HSCT to evaluate the extent of iron deposition in children. Timely iron chelation therapy before HSCT reduces the extent of iron deposition, further reduces transplant-related complications and increases event-free and overall survival.

## Data Availability

The original contributions presented in the study are included in the article/Supplementary Material, further inquiries can be directed to the corresponding author/s.
